# You Can’t Just Hope for the Best: VA and Non-VA Home-Based Long-Term Care in Puerto Rico Following Hurricane Maria

**DOI:** 10.1093/geroni/igaa057.1981

**Published:** 2020-12-16

**Authors:** Leah Haverhals

**Affiliations:** Department of Veterans Affairs, Denver, Colorado, United States

## Abstract

This research describes how home-based long-term care settings in Puerto Rico, connected to the United States Department of Veterans Affairs (VA) and in non-VA settings, prepared for and secured the safety and wellbeing of elderly and disabled persons during and after Hurricane Maria, which struck Puerto Rico on September 20, 2017. I collected data via in-person interviews, home visits, and field observations between January-March 2019. Guided by a social vulnerability and health model, I interviewed a multitude of people connected to and/or caring for elderly and disabled populations in these settings. Results emphasize importance of disaster preparedness, incorporating lessons learned from hardships, and how Puerto Rico’s colonial status and economic realities influenced recovery. VA’s interconnected nature provided a stronger support network compared to non-VA settings that were often independently or family run. Regardless of setting, the resilience and collaborative spirit of Puerto Ricans proved instrumental in recovery and disaster management.

